# Fusion of medical imaging and electronic health records using deep learning: a systematic review and implementation guidelines

**DOI:** 10.1038/s41746-020-00341-z

**Published:** 2020-10-16

**Authors:** Shih-Cheng Huang, Anuj Pareek, Saeed Seyyedi, Imon Banerjee, Matthew P. Lungren

**Affiliations:** 1 Department of Biomedical Data Science, Stanford University, Stanford, USA; 2 Center for Artificial Intelligence in Medicine & Imaging, Stanford University, Stanford, USA; 3 Department of Radiology, Stanford University, Stanford, USA; 4 Department of Biomedical Informatics, Emory University, Atlanta, USA; 5 Department of Radiology, Emory University, Atlanta, USA

**Keywords:** Data integration, Machine learning, Medical imaging

## Abstract

Advancements in deep learning techniques carry the potential to make significant contributions to healthcare, particularly in fields that utilize medical imaging for diagnosis, prognosis, and treatment decisions. The current state-of-the-art deep learning models for radiology applications consider only pixel-value information without data informing clinical context. Yet in practice, pertinent and accurate non-imaging data based on the clinical history and laboratory data enable physicians to interpret imaging findings in the appropriate clinical context, leading to a higher diagnostic accuracy, informative clinical decision making, and improved patient outcomes. To achieve a similar goal using deep learning, medical imaging pixel-based models must also achieve the capability to process contextual data from electronic health records (EHR) in addition to pixel data. In this paper, we describe different data fusion techniques that can be applied to combine medical imaging with EHR, and systematically review medical data fusion literature published between 2012 and 2020. We conducted a systematic search on PubMed and Scopus for original research articles leveraging deep learning for fusion of multimodality data. In total, we screened 985 studies and extracted data from 17 papers. By means of this systematic review, we present current knowledge, summarize important results and provide implementation guidelines to serve as a reference for researchers interested in the application of multimodal fusion in medical imaging.

## Introduction

The practice of modern medicine relies heavily on synthesis of information and data from multiple sources; this includes imaging pixel data, structured laboratory data, unstructured narrative data, and in some cases, audio or observational data. This is particularly true in medical image interpretation where substantial clinical context is often essential to provide diagnostic decisions. For example, it has repeatedly been shown that a lack of access to clinical and laboratory data during image interpretation results in lower performance and decreased clinical utility for the referring provider^1,2^. In a survey of radiologists, the majority (87%) stated that clinical information had a significant impact on interpretation^3^. The importance of clinical context for accurate interpretation of imaging data is not limited to radiology; instead many other imaging-based medical specialties such as pathology, ophthalmology, and dermatology, also rely on clinical data to guide image interpretation in practice^4–6^. Pertinent and accurate information regarding the current symptoms and past medical history enables physicians to interpret imaging findings in the appropriate clinical context, leading to a more relevant differential diagnosis, a more useful report for the physicians, and optimal outcome for the patient.

In the current digital era, the volume of radiological imaging exams is growing. To meet this increased workload demand, an average radiologist may have to interpret an image every 3–4 s over an 8-h workday which contributes to fatigue, burnout, and increased error-rate^7^. Deep learning in healthcare is proliferating due to the potential for successful automated systems to either augment or offload cognitive work from busy physicians^8–10^. One class of deep learning, namely convolutional neural networks (CNN) has proven very effective for image recognition and classification tasks, and are therefore often applied to medical images. Early applications of CNNs for image analysis in medicine include diabetic retinopathy, skin cancer, and chest X-rays^11–18^. Yet, these models consider only the pixel data as a single modality for input and cannot contextualize other clinical information as would be done in medical practice, therefore may ultimately limit clinical translation.

As an example consider the “simple” task in radiology of identifying pneumonia on a chest radiograph, something that has been achieved by many investigators training deep learning models for automated detection and classification of pathologies on chest X-rays^19,20^. Yet without clinical context such as patient history, chief complaint, prior diagnoses, laboratory values, such applications may ultimately have limited impact on clinical practice. The imaging findings on chest X-rays consistent with pneumonia, despite having imaging features that can generally differentiate alternative diagnoses, are nonspecific and accurate diagnosis requires the context of clinical and laboratory data. In other words, the chest X-ray findings that suggest pneumonia would be accurate in one person with fever and an elevated white blood cell count but in another patient without those supporting clinical characteristics and laboratory values, similar imaging finding may instead represent other etiologies such as atelectasis, pulmonary edema, or even lung cancer. There are countless examples across different medical fields in which clinical context, typically in the form of structured and unstructured clinical data from the electronic health record (EHR), is critical for accurate and clinically relevant medical imaging interpretation. As with human physicians, automated detection and classification systems that can successfully utilize both medical imaging data together with clinical data from the EHR, such as patient demographics, previous diagnoses and laboratory values, may lead to better performing and more clinically relevant models.

Multimodal deep learning models that can ingest pixel data along with other data types (fusion) have been successful in applications outside of medicine, such as autonomous driving and video classification. As an example, a multimodal fusion detection system for autonomous vehicles, that combines visual features from cameras along with data from Light Detection and Ranging (LiDAR) sensors, is able to achieve significantly higher accuracy (3.7% improvement) than a single-modal CNN detection model^21^. Similarly, a multimodal social media video classification pipeline leveraging both visual and textual features increased the classification accuracy to 88.0%, well above single modality neural networks such as Google’s InceptionV3 which reached an accuracy of 76.4% on the same task^22^. The improvements in performance for these efforts not only echo the justification in medical applications, leveraging fusion strategies for medical imaging is also primarily motivated by the desire to integrate complementary contextual information and overcome the limitation of image-only models.

**Fig. 1:**
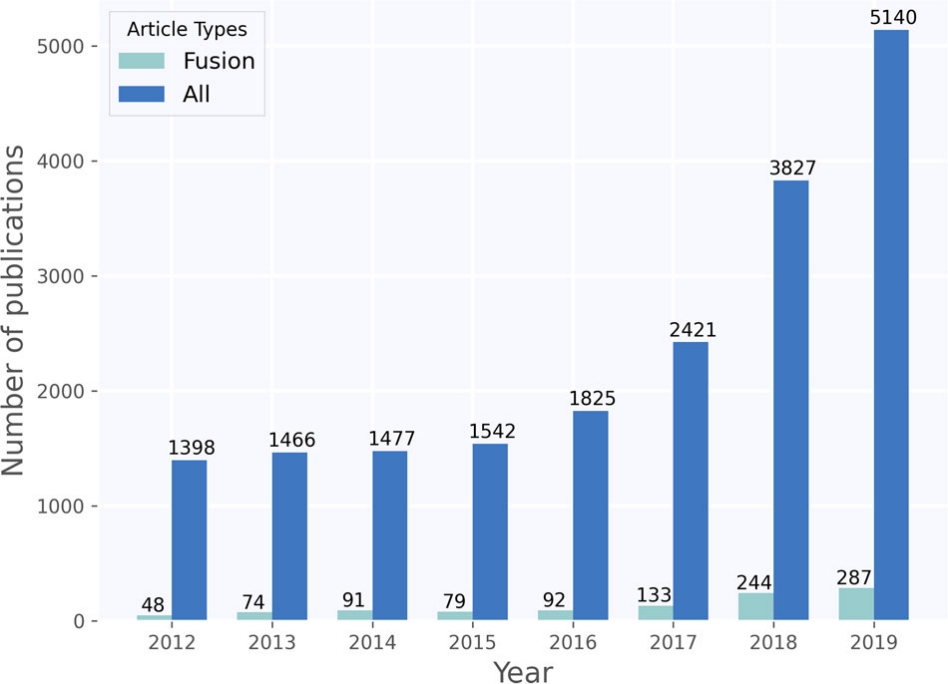
Timeline of publications in deep learning for medical imaging. Timeline showing growth in publications on deep learning for medical imaging, found by using the same search criteria on PubMed and Scopus. The figure shows that fusion has only constituted a small, but growing, subset of medical deep learning literature.

The recent medical imaging literature shows a similar trend where both EHR and pixel data are leveraged in a “fusion-paradigm” for solving complex tasks which cannot readily be tackled by a single modality (Fig. 1). The new fusion paradigm covers a wide range of methodologies and techniques with varying terms and model architectures that have not been studied systematically. The purpose of this review paper is to present a comprehensive analysis of deep learning models that leverage multiple modalities for medical imaging tasks, define and consolidate relevant terminology, and summarize the results from state-of-the-art models in relevant current literature. We hope this review can help inform future modeling frameworks and serve as a reference for researchers interested in the application of multimodal fusion in medical imaging.

### Terminology and strategies in fusion

Data fusion refers to the process of joining data from multiple modalities with the aim of extracting complementary and more complete information for better performing machine learning models as opposed to using a single data modality.

**Fig. 2:**
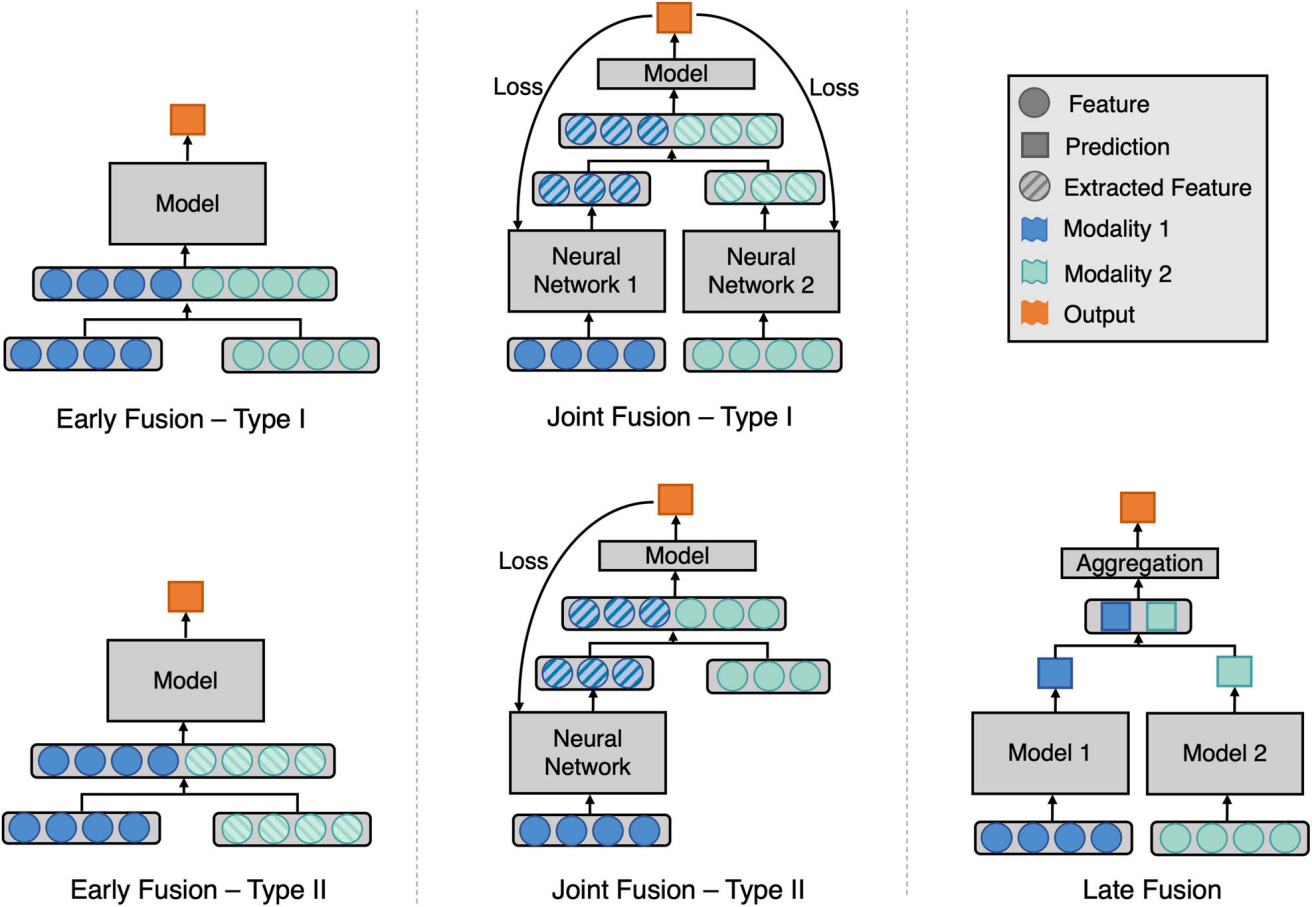
Fusion strategies using deep learning. Model architecture for different fusion strategies. Early fusion (left figure) concatenates original or extracted features at the input level. Joint fusion (middle figure) also joins features at the input level, but the loss is propagated back to the feature extracting model. Late fusion (right figure) aggregates predictions at the decision level.

Figure 2 illustrates the three main different fusion strategies, namely early, joint, and late fusion. Here we define and describe each fusion strategy in detail:

**Early fusion**^23^, commonly known as feature level fusion, refers to the process of joining multiple input modalities into a single feature vector before feeding into one single machine learning model for training (Fig. 2 Early Fusion). Input modalities can be joined in many different ways, including concatenation, pooling or by applying a gated unit^23,24^. Fusing the original features represents early fusion type I, while fusing extracted features, either from manual extraction, imaging analysis software or learned representation from another neural network represents early fusion type II. We consider predicted probabilities to be extracted features, thus fusing features with predicted probabilities from different modalities is also early fusion type II.

**Joint fusion** (or intermediate fusion) is the process of joining learned feature representations from intermediate layers of neural networks with features from other modalities as input to a final model. The key difference, compared to early fusion, is that the loss is propagated back to the feature extracting neural networks during training, thus creating better feature representations for each training iteration (Fig. 2 Joint Fusion). Joint fusion is implemented with neural networks due to their ability to propagate loss from the prediction model to the feature extraction model(s). When feature representations are extracted from all modalities, we consider this joint fusion type I. However, not all input features require the feature extraction step to be defined as joint fusion (Fig. 2 Joint Fusion—Type II).

**Late fusion**^23^ refers to the process of leveraging predictions from multiple models to make a final decision, which is why it is often known as decision-level fusion (Fig. 2 Late Fusion). Typically, different modalities are used to train separate models and the final decision is made using an aggregation function to combine the predictions of multiple models. Some examples of aggregation functions include: averaging, majority voting, weighted voting or a meta-classifier based on the predictions from each model. The choice of the aggregation function is usually empirical, and it varies depending on the application and input modalities.

## Results

**Fig. 3:**
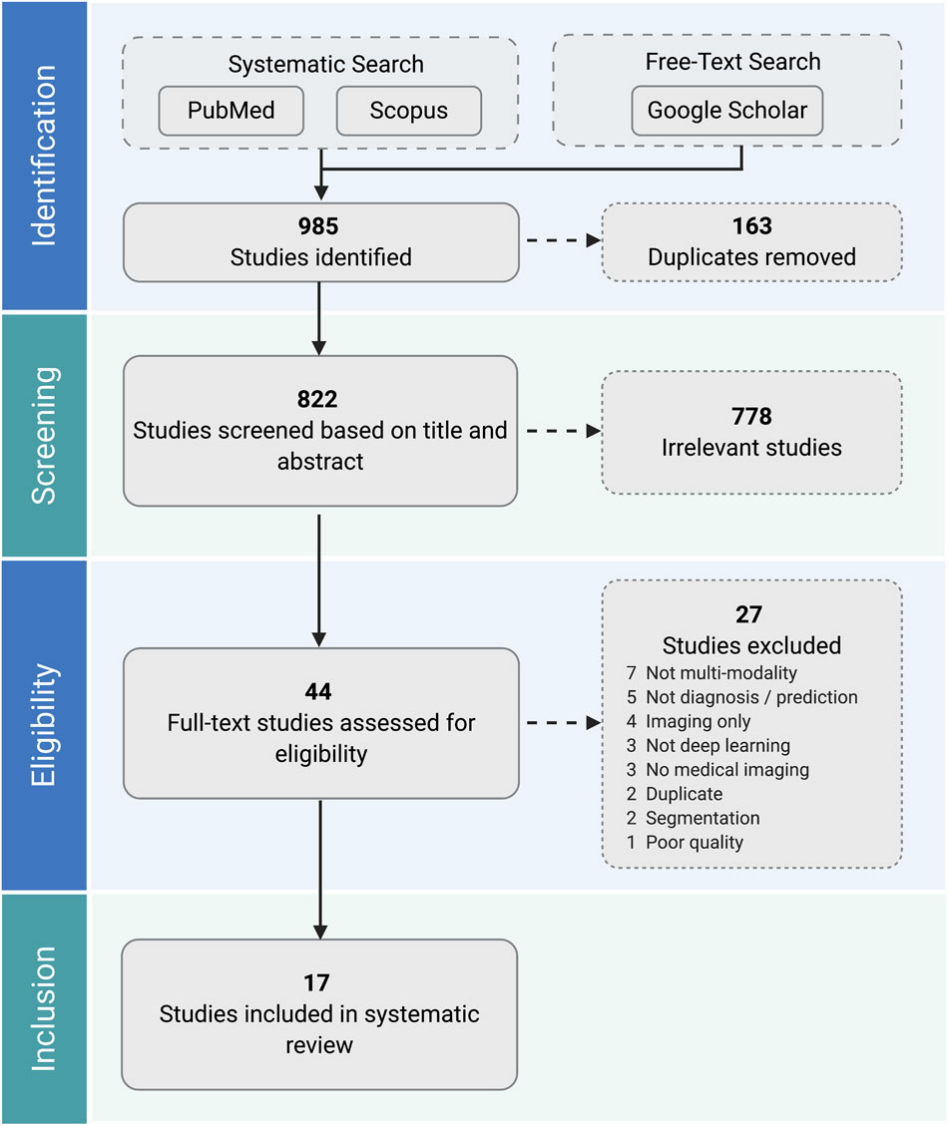
PRISMA flowchart of the study selection process. Two authors independently screened all records for eligibility. Seventeen studies were included in the systematic review.

A total of 985 studies were identified through our systematic search. After removing duplicates and excluding studies based on title and abstract using our study selection criteria (see Methods), 44 studies remained for full-text screening. A total of 17 studies fulfilled our eligibility criteria and were included for systematic review and data extraction. The studies were in English except for a single paper in Chinese. Figure 3 presents a flowchart of the study screening and selection process and Table 1 displays the included studies and extracted data.

**Table 1:** Overview of studies included in the systematic review.

Fusion strategy	Year	Author	Clinical domain	Outcome	Fusion details	Input: medical imaging	Input: non-imaging data	Number of samples	Model performance
Early	2017	Thung et al.^25^	Neurology	Diagnosis of Alzheimer’s disease	Software or manually extracted features	PET, MRI	Patient data (age, sex, education) Genetic data (APOE4)	805	Fusion: 63.6% Accuracy MRI + PET: 61.1% Accuracy MRI: 58.0% Accuracy
Early	2018	An et al.^35^	Ophthalmology	Glaucoma classification	Software or manually extracted features	Optical coherence tomography, Laser speckle flowgraphy	Patient data (age, sex, spherical equivalent)	163	Fusion: 87.8% Accuracy
Early (multi-stage)	2018	Bhagwat et al.^33^	Neurology	Prediction of clinical symptom trajectories in Alzheimer’s disease	Software or manually extracted features	MRI	Patient data (age, clinical scores) Genetic data (APOE4)	1302	Fusion: 0.99 AUROC MRI: 0.83 AUROC Clinical Attributes: 0.97 AUROC
Early	2018	Kharazmi et al.^26^	Dermatology	Basal cell carcinoma detection	CNN extracted features	Dermoscopic images	Patient data (age, sex, elevation, lesion location, lesion size)	1191	Fusion: 91.1% Accuracy Dermoscopic Images: 84.7% Accuracy Patient Profile: 75.6% Accuracy
Early	2018	Liu et al.^34^	Neurology	Prediction of rupture risk in anterior communicating artery aneurysms	Software or manually extracted features	CT	Patient data (age, sex, hypertension, smoking habits)	594	Fusion: 0.928 AUROC
Early	2018	Liu et al.^30^	Radiology	Bone age assessment	CNN extracted features	X-ray	Patient data (age, sex)	11,858	Fusion: 0.455 Mean Absolute Error
Early	2018	Yap et al.^27^	Dermatology	Classification of skin lesion and detection of melanoma	CNN extracted features	Macroscopic images, Dermoscopic images	Patient data (age, sex, body location)	2917	Fusion: 0.888 AUROC Macroscopic + Dermoscopic Images: 0.888 AUROC Patient metadata: 0.810 AUROC
Early	2019	Hyun et al.^32^	Radiology/Oncology	Lung cancer	Software or manually extracted features	PET/CT	Patient data (age, sex, tumor size, smoking status)	396	Fusion: 0.859 AUROC
Early	2019	Li et al.^28^	Neurology	Prediction of Alzheimer’s disease	CNN extracted features	MRI	Assessments (Alzheimer’s Disease Assessment Scale-Cognitive subscale, Rey Auditory Verbal Learning Test, Functional Assessment Questionnaire, and Mini-Mental State Examination) Patient data (age, gender, education, APOE4)	822	Fusion: 0.901 C-index Cognitive data: 0.896 C-index
Early	2019	Nie et al.^31^	Radiology/Oncology	Prediction of survival time for brain tumor patients	CNN extracted features	MRI	Patient data (age, tumor size, histological type)	93	Fusion: 90.66% Accuracy MRI: 81.04% Accuracy Demographics and tumor features: 62.96% Accuracy
Early	2020	Purwar et al.^29^	Hematology	Detection of microcytic hypochromia	CNN extracted features	Images of Red Blood Cells	Blood test (complete blood count, haematocrit, HCT, MCV, MCH, MCHC, RDW, hemoglobin A1 hemoglobin A2, hemoglobin F, Mentzer index)	20	Fusion: 1.00 AUROC Images: 0.88 AUROC Blood count features: 0.93 AUROC
Joint*	2018	Kawahara et al.^39^	Dermatology	Melanoma classification and binary classification of each of the seven-point checklist for melanoma	Multimodal multi-task	Dermoscopic Images, Clinical Images	Patient data (sex, lesion location)	1011	Fusion: 73.7% Accuracy Dermoscopic Images: 72.5% Accuracy Clinical Images: 64.1% Accuracy
Joint	2018	Spasov et al.^36^	Neurology	Prediction of Alzheimer’s disease	–	MRI	Patient data (age, sex, race, education, biofluids) Genetic data (APOE4) Assessments (Clinical Dementia rating [CSRSB], Alzheimer’s disease assessment scale, Rey auditory verbal learning test)	376	Fusion: 1.00 AUROC
Joint	2019	Yala et al.^37^	Radiology/Oncology	Breast cancer risk prediction	–	Mammograms	Patent data (age, weight, height, menarche age, menopausal status, detailed family history of breast and ovarian cancer, BRCA mutation status, history of atypical hyperplasia, history of lobular carcinoma in situ, and breast density)	88,994	Fusion: 0.70 AUROC Mammograms: 0.68 AUROC Risk scores: 0.67 AUROC
Joint Late	2019	Yoo et al.^38^	Neurology	Predicting status conversion to multiple sclerosis within two years	CNN extracted feature Averaging (Late)	MRI	Patient data (sex, extended disability status scale, uni- vs. multifocal clinically isolated syndrome (CIS) at onset, location of initial CIS event)	140	Joint Fusion: 0.746 AUROC Late Fusion: 0.724 AUROC MRI: 0.718 AUROC Clinical Features: 0.679 AUROC
Late	2018	Qiu et al.^41^	Neurology	Mild cognitive impairment (MCI)	Majority voting	MRI	Assessments (Mini Mental State Examination (MMSE), Wechsler Memory Scale Logical Memory (LM) test)	386	Fusion: 90.9% Accuracy MRI: 83.1% Accuracy MMSE: 84.3% Accuracy LM: 89.1% Accuracy
Late	2018	Reda et al.^40^	Radiology/Oncology	Prostate cancer diagnosis	Meta classifier	MRI	PSA blood test	18	Fusion: 94.4% Accuracy MRI: 88.89% Accuracy PSA: 77.78% Accuracy

The table provides an overview of all studies included in the systematic review including fusion strategy and data extracted from each study.

### Early fusion

The majority of the studies that remained after our full-text screening (11/17) used early fusion to join the multimodal input. Thung et al.^25^ conducted image-image fusion of PET and MRI images using a joint fusion approach, but since they concatenated clinical and imaging features into one single feature vector before feeding into their neural network, we categorized their approach as early fusion. Six out of eleven early fusion studies extracted features from medical imaging using a CNN (Table 1). Four out of the six studies that applied neural networks for feature extraction simply concatenated the extracted imaging features with clinical features for their fusion strategy^26–29^. The remaining two studies by Liu et al.^30^ and Nie et al.^31^ applied dimensionality reduction techniques before concatenating the features. Five studies used software generated and/or manually extracted features from medical imaging before fusing with clinical data. Software-based feature extraction included radiomics features such as skewness and kurtosis^32^ or volume and thickness quantification of the regions of interest^25,33^. Manually extracted features included radiological assessments such as size, angle, and morphology of anatomical structures^34^. Out of these five studies, two applied feature selection strategies to reduce the feature dimension and improve predictive performance. The employed feature selection strategies included a rank-based method using Gini coefficients^32^, a filter-based method based on mutual information of the features^35^, and a genetic-algorithm based method^35^. Seven of the early fusion studies compared the performance of their fusion models against single modality models (Table 1). Six of these studies showed an improvement in performance when using fusion^25,26,28,29,31,33^, and the remaining one achieved the same performance but reduced standard deviation^27^, alluding to a model with better stability.

### Joint fusion

Joint fusion was used in four out of the seventeen studies. Spasov et al.^36^, Yala et al.^37^, and Yoo et al.^38^ implemented CNNs to learn image features and fused these feature representations with clinical features before feeding them into a feed-forward neural network. Spasov et al. and Yala. et al. both used simple concatenation to fuse the learned imaging and clinical features. To cater to the differences between the dimensionality and dynamic range between the imaging and clinical features, Yoo et al. replicated and scaled their clinical features before fusion and they observed improvements in performances. Kawahara et al.^39^ also used CNNs as feature extractors for imaging modalities but experimented with a unique multimodal multi-task loss function that considers multiple combinations of the input modalities. The predicted probabilities of these multi-task outputs were aggregated for prediction, but we do not consider this late fusion since the probabilities were not from separate models. Kawahara et al., Yala et al. and Yoo et al. reported an improvement in performance using fusion compared to image-only models (Table 1). Yoo et al. further compared their joint fusion model to a late fusion model and achieved a 0.02 increase in Area Under Receiver Operating Characteristic Curve (AUROC).

### Late fusion

Late fusion was used in three out of the seventeen included studies (Table 1). Each of the three late fusion papers applied a different type of aggregation strategy. Yoo et al.^38^ took the mean of the predicted probabilities from two single modality models as the final prediction. Reda et al.^40^ built another classifier using the single modality models’ prediction probabilities as inputs. Qiu et al.^41^ trained three independent imaging models that took as input a single MRI slice, each from a specific anatomical location. Max, mean and majority voting were applied to aggregate predictions from the three imaging models. The results from the three aggregation methods were combined again by majority voting before another round of late fusion with the clinical models. All late fusion models showed improvements in performances when compared to models that used only single modalities.

## Discussion

The purpose of this review is to aggregate the collective knowledge of prior work applying multimodal deep learning fusion techniques that combine medical imaging with clinical data. We propose consistent terminology for multimodal fusion techniques and categorize prior work by fusion strategy. Overall, we found that multimodality fusion models generally led to increased accuracy (1.2–27.7%) and AUROC (0.02–0.16) over traditional single modality models for the same task. However, no single fusion strategy consistently led to optimal performance across all domains. Since our literature review shows that additional patient information and clinical context can result in better model performance, and fusion methods better replicate the human expert interpretation workflow, it is recommended to always experiment with fusion strategies when multimodal data is available.

The deep learning fusion models reviewed represent a spectrum of medical applications ranging from radiology^31^ to hematology^29^. For example, fusion strategies were often applied to the diagnosis and prediction of Alzheimer’s disease^25,28,33,36,41^. In clinical practice, neither imaging nor clinical data alone are sufficient for the diagnosis of Alzheimer’s disease. Leveraging deep learning fusion techniques consistently showed improvements in performance for Alzheimer’s disease diagnosis, while physicians struggle with accurate and reliable diagnostics even when multimodality is present, as proven by histopathological correlation^42^. This highlights the importance and utility of multimodal fusion techniques in clinical applications.

Fusion approaches in other less complex clinical applications also improved performance over single modality models, even those in which single modality models have been widely reported to achieve high performance, such as pixel-based models for automated skin cancer detection^43^. While the fusion approach varied widely, the consistent improvement in reported performance across a wide variety of clinical use cases suggests that model performance based on single-modal data may not represent state of the art for a given application when multimodal data are not considered.

The complexity of the non-imaging data in multimodal fusion work was limited, particularly in the context of available feature-rich and time-series data in the EHR. Instead, most studies focused primarily on basic demographic information such as age and gender^25,27,39^, a limited range of categorical clinical history such as hypertension or smoking status^32,34^ or disease-specific clinical features known to be strongly associated with the disease of interest such as APOE4 for Alzheimer’s^25,28,33,36^ or PSA blood test for prediction of prostate cancer^40^. While selecting features known to be associated with disease is meaningful, future work may further benefit from utilizing large volumes of feature-rich data, as seen in fields outside medicine such as autonomous driving^44,45^.

### Implementation guidelines for fusion models

In most applications early fusion was used as the first attempt for multimodal learning, a straightforward approach that does not necessarily require training multiple models. However, when the input modalities are not in the same dimensions, which is typical when combining clinical data represented in 1D with imaging data in 2D or 3D, then high-level imaging features must be extracted as a 1D vector before fusing with the 1D clinical data. There were a variety of strategies used to accomplish this; including using manually extracted imaging features or software-generated features^25,32–35^. It is worth noting, that unless there is a compelling reason for using such an approach, outputs from linear layers of a CNN are usually effective feature representations of the original image^28,29,31^. This is because learned features representations often result in much better task-specific performance than can be obtained with manual or software extracted features^46^. Based on the reviewed papers, early fusion consistently improved performance over single modality models, and is supported by this review as an initial strategy to fuse multimodal data.

When using CNNs to extract features from imaging modalities, the same CNNs can also be used in joint fusion. However, joint fusion is implemented using neural networks which can be a limitation especially with smaller datasets better suited for traditional machine learning models. For example, if there are disproportionately few samples relative to the number of features in the dataset or if some of the input features are sparsely represented, early or late fusion is preferred because they can be implemented with traditional machine learning algorithms (e.g., Lasso and ElasticNet^47^) that are better suited for this type of data^48^. Nevertheless, joint and early fusion neural networks are both able to learn shared representations, making it easier for the model to learn correlations across modalities, thereby resulting in better performance^49^. Studies have also shown that fusing highly correlated features in earlier layers and less correlated features in deeper layers improve model performance^50,51^. In addition, we suspect that joint fusion models have the potential to outperform other fusion strategies, as the technique iteratively updates its feature representations to better complement each modality through simultaneous propagation of the loss to all feature extracting models. Yet to date, there is insufficient evidence to systematically assess this effect in fusion for medical imaging and is an important area for future exploration.

When signals from different modalities do not complement each other, that is to say input modalities separately inform the final prediction and do not have inherent interdependency, then trying a late fusion approach is preferred. This is chiefly because when feature vectors from multiple modalities are concatenated, such as in early and joint fusion, high-dimensional vectors are generated which can be difficult for machine learning models to learn without overfitting, unless a large number of input samples are available. This is the so-called “curse of dimensionality” in machine learning^52,53^. Late fusion mitigates this problem by utilizing multiple models that are each specialized on a single modality, thus limiting the input feature vector size for each model. For example, the quantitative result of a Mini Mental State Examination and the pixel data obtained from a brain MRI (e.g., Qiu et al.^41^) are largely independent data, and would therefore be suitable candidates for input into late fusion models.

Furthermore, in the common real-world scenario of missing or incomplete data, i.e. some patients have only clinical data available but no imaging data or vice-versa, late fusion retains the ability to make predictions. This is because late fusion employs separate models for separate modalities, and aggregation functions such as majority voting and averaging can be applied even when predictions from a modality is missing. When the different input modalities have very different numbers of features, predictions might be overly influenced by the most feature-rich modality (e.g., Reda et al.^40^). Late fusion is favorable in this scenario as it considers each modality separately. Yoo et al.^38^ also showed that repeating or scaling the modality that has fewer features before fusion achieved a boost in the model’s performance. Nonetheless, joint fusion can also be tuned to mitigate the difference in number of features, by setting feature producing linear layers of the feature extraction model to output a similar number of features as the other modalities. Our recommendations are summarized in Table 2.

**Table 2:** Properties and benefits of different fusion strategies.

	Early	Joint	Late
Able to make predictions when not all modalities are present	×	×^a^	✓
Able to model interactions between features from different modalities	✓	✓	×
Able to learn more compatible features from each modality	×	✓	×
Does not necessarily require a large amount of training data	×	×	✓
Does not require training multiple models	✓^b^	✓	×
Does not necessarily require meticulous designing efforts	✓	×	✓
Flexibility to join input at different levels of abstraction	×	✓	×

Different properties and benefits for each fusion strategy.

^a^Specialized joint fusion architecture such as Kawahara et al.’s multi-modal multi-task model is capable of handling missing data.

^b^Early fusion requires training of multiple models when the imaging features are extracted using CNN.

Ideally, researchers want to first build and optimize single modality models to dually serve as baseline models and provide inputs to fusion models. Multiple fusion strategies can then be implemented to compare model performance and guide subsequent fusion experiments. Since better performance is consistently achieved with multimodal fusion techniques, routine best practice should include reporting of the systematic investigation of various fusion strategies in addition to deep learning architectures and hyperparameters.

### Limitations

We devised our search string to only consider papers after 2012. This constitutes a limitation as we excluded earlier papers that applied fusion using traditional machine learning techniques or simple feed-forward neural networks. Publication bias is an important limitation since positive results can be disproportionately reported in the published literature, which may have the aggregate effect of overrepresenting the advantages of fusion techniques. Furthermore, using our study selection criteria, we only looked at fusion techniques applied to clinical prediction and diagnosis, but we recognize that fusion can be applied to other interesting medical tasks such as segmentation and registration.

As the included studies investigate different objectives, use different input modalities, report different performance metrics, and not all papers provide confidence bounds, we are not able to aggregate or statistically compare the performance gains in a meta-analysis. In addition, the reported metrics cannot always be considered valid, since some studies didn’t use an independent test-set for an unbiased performance estimate^29,40^. The limited number of studies per medical field and the heterogeneity of each study also makes it difficult to compare the studies qualitatively. A few studies implemented fusion in unconventional ways, which may introduce subjectivity when we classify each study into early, late, and joint fusion.

### Future research

This systematic review found that multimodal fusion in medicine is a promising yet nascent field that complements the clinical practice of medical imaging interpretation across all disciplines. We have defined and summarized key terminology, techniques, and evaluated the state of the art for multimodal fusion in medical imaging, honing in on key insights and unexplored questions to guide task and modality-specific strategies. The field of multimodal fusion for deep learning in medical imaging is expanding and novel fusion methods are expected to be developed. Future work should focus on shared terminology and metrics, including direct evaluation of different multimodal fusion approaches when applicable. We found that multimodal fusion for automated medical imaging tasks broadly improves the performance over single modality models, and further work may discover additional insights to inform optimal approaches.

## Methods

This systematic review was conducted based on the PRISMA guidelines^54^.

### Search strategy

A systematic literature search was implemented in PubMed and Scopus under the supervision of a licensed librarian. The key search terms included a combination of the three major themes: ‘deep learning’, ‘multimodality fusion’, and ‘medical imaging’. Terms for segmentation, registration, and reconstruction were used as exclusion criteria in the search. The search encompassed papers published between 2012 and 2020. This range was considered appropriate due to the rise in popularity in applying CNN on medical images since the 2012 ImageNet challenge. The complete search string for both databases is provided in Supplementary Methods. For potentially eligible studies cited by articles already included in this review, additional targeted free-text searches were conducted on Google Scholar if they did not appear in Scopus or PubMed.

We included all research articles in all languages that applied deep learning models for clinical outcome prediction or diagnosis using a combination of medical imaging modalities and EHR data. Studies specific to deep learning were included rather than the broader field of machine learning because deep learning has consistently shown superior performance in image-related tasks. We selected only studies that fused medical imaging with EHR data since, unlike image-image fusion, this is an exciting new technique that effectively merges heterogeneous data types and adds complementary rather than overlapping information to inform prediction and diagnosis. We defined medical imaging modalities as any type of medical images used in clinical care. Studies that used deep learning only for feature extractions were also included for our review. We excluded any study that combined extracted imaging features with the original imaging modality, as we still considered this a single modality. Articles that fused multimodal data for segmentation, registration or reconstruction were also excluded due to our criteria for outcome prediction and diagnosis. Articles from electronic preprint archives such as ArXiv were excluded in order to ensure only papers that passed peer-review were included. Lastly, papers with poor quality that hindered our ability to meaningfully extract data were also excluded.

### Study selection

The Covidence software (www.covidence.org) was used for screening and study selection. After removal of duplicates, studies were screened based on title and abstract, and then full-texts were obtained and assessed for inclusion. Study selection was performed by two independent researchers (S.-C.H. and A.P.), and disagreements were resolved through discussion. In cases where consensus could not be achieved a third researcher was consulted (I.B.).

### Data extraction

For benchmarking the existing approaches we extracted the following data from each of the selected articles: (a) fusion strategy, (b) year of publication, (c) authors, (d) clinical domain, (e) target outcome, (f) fusion details, (g) imaging modality, (h) non-imaging modality, (i) number of samples, and (j) model performance (Table 1). We classified the specific fusion strategy based on the definitions in the section “Terminology and strategies in fusion”. The number of samples reported is the full data-size including training, validation and testing data. For classification tasks we extracted AUROC whenever this metric was reported, otherwise we extracted accuracy. When the article contained several experiments, metrics from the experiment with the best performing fusion model were extracted. These items were extracted to enable researchers to find and compare current fusion studies in their medical field or input modalities of interest.

## Supplementary information

Supplementary Information

## Data Availability

The authors declare that all data supporting the findings of this study are available within the paper and its Supplementary information files.
