# Supplementary material for: Fusion of medical imaging and electronic health records using deep learning: a systematic review and implementation guidelines
Source: NPJ Digit Med. 2020 Oct 16;3:136. doi: 10.1038/s41746-020-00341-z (PMC7567861; doi:10.1038/s41746-020-00341-z)
Supplement: Supplementary file 1 — Supplementary Information [file 41746_2020_341_MOESM1_ESM.pdf]

Supplementary Methods

Systematic search string on PubMed

(("neural networks, computer"[mh] OR "neural network"[tw] OR "neural networks"[tw] OR "Deep learning"[tw] OR "hierarchical learning"[tw] OR "Recurrent neural network*"[tw] OR "Convolutional neural network"[tw] OR “autoencoder”[tw]) AND ("multimodal imaging"[mh] OR "multimodal"[tw] OR "hybrid"[All Fields] OR "late fusion"[tw] OR "joint fusion"[tw] OR "early fusion"[tw] OR "feature fusion"[tw]) AND ("Diagnosis"[mh] OR "Diagnosis, Computer-Assisted"[mh] OR "Diagnosis"[tw] OR "diagnostic imaging"[mh] OR "CT Scan"[tw] OR "CT Scans"[tw] OR "PET Scan"[tw] OR "PET Scans"[tw] OR "MRI"[tw] OR "X-Ray"[tw] OR "Mammogram"[tw] OR "ultrasound"[tw] OR "endoscopy"[tw])) NOT ("Registration"[tw] OR "Segmentation"[tw] OR "Reconstruction"[tw]) NOT ("Registration"[tw] OR "Segmentation"[tw] OR "Reconstruction"[tw]) AND "Journal Article"[ptyp] AND ("2012/09/30"[PDAT] : "2019/12/31"[PDAT])

Systematic search string on Scopus

((TITLE-ABS-KEY ("Deep Learning") OR TITLE-ABS-KEY ("hierarchical learning") OR TITLE-ABS-KEY ("Recurrent neural network*") OR TITLE-ABS-KEY ("Convolutional neural network*") OR TITLE-ABS-KEY ("Deep neural network*") OR TITLE-ABS-KEY ("Neural Network*") OR TITLE-ABS-KEY ("autoencoder”)) AND DOCTYPE (ar) AND PUBYEAR > 2011 AND PUBYEAR < 2020) AND ((TITLE-ABS-KEY ("Multimodal" OR "Hybrid" OR "Late fusion" OR "Early fusion" OR "Joint fusion" OR "Feature fusion")) AND DOCTYPE (ar) AND PUBYEAR > 2011 AND PUBYEAR < 2020) AND ((TITLE-ABS-KEY ("Diagnosis" OR "Diagnostic" OR "CT Scan*" OR "PET Scan*" OR "MRI Scan*" OR "X Ray*" OR "Mammogram*" OR "Ultrasound" OR "Endoscopy")) AND DOCTYPE (ar) AND PUBYEAR > 2011 AND PUBYEAR < 2020) AND NOT ((TITLE-ABS-KEY ("registration" OR "segmentation*" OR "reconstruction*")) AND DOCTYPE (ar) AND PUBYEAR > 2011 AND PUBYEAR < 2020)
